# Nutritional Security in Drylands: Fast-Track Intra-Population Genetic Improvement for Grain Iron and Zinc Densities in Pearl Millet

**DOI:** 10.3389/fnut.2019.00074

**Published:** 2019-05-24

**Authors:** Mahalingam Govindaraj, Kedar Nath Rai, Anand Kanatti, Aluri Sambasiva Rao, Harshad Shivade

**Affiliations:** Crop Improvement, International Crops Research Institute for the Semi-Arid Tropics (ICRISAT), Patancheru, India

**Keywords:** biofortification, iron, open-pollinated variety, pearl millet, recurrent selection, X-ray fluorescence spectrometer, zinc

## Abstract

Considering the pervasive malnutrition caused by micronutrients, particularly those arising from the deficiencies of iron (Fe) and zinc (Zn), the primary focus of research in pearl millet is on biofortifying the crop with these two minerals. Pearl millet is a highly cross-pollinated crop where open-pollinated varieties (OPVs) and hybrids are the two distinct cultivar types. In view of the severe deficiency of Fe and Zn in Asia and Africa where this crop is widely consumed, crop biofortification holds a key role in attenuating this crisis. The present study included three OPVs previously identified for high-Fe and Zn density to assess the magnitude of variability and test the effectiveness of intra-population improvement as a fast-track selection approach. Large variability among the S_1_ progenies was observed in all three OPVs, with the Fe varying from 31 to 143 mg kg^−1^ and Zn varying from 35 to 82 mg kg^−1^. Progeny selection was effective for Fe density in all three OPVs, with up to 21% selection response for Fe density, and up to 10% selection response in two OPVs for Zn density, for which selection was made as an associated trait. Selection for Fe density had no adverse effect on grain yield and other agronomic traits. These results suggest that effective selection for Fe density in OPVs and composites can be made for these micronutrients and selection for Fe density is highly associated with the improvement of Zn density as well. These genetic changes can be achieved without compromising on grain yield and agronomic traits. Such improved versions could serve as essentially-derived varieties for immediate cultivation and also serve as potential sources for the development of parental lines of hybrids with elevated levels of Fe and Zn density. Therefore, fast-track breeding is essential to produce biofortified breeding pipelines to address food-cum-nutritional security.

## Introduction

Pearl millet (*Pennisetum glaucum* (L.) R. Br.) is a highly cross-pollinated crop with more than 85% outcrossing (1). This floral system provides for open-pollinated varieties (OPVs) and hybrids as the two broad cultivar options. Pearl millet is cultivated in around 26 million ha, primarily for grain production, but it is also valued for its stover as a source of livestock fodder in the arid and semi-arid tropical regions of Africa and Asia, with India having the largest area of about 10 million ha (2). While the development of OPVs continues to be the thrust area of research in Africa, the development of hybrids is the primary focus in India, with OPVs as the second priority. OPVs are genetically heterogeneous and phenotypically variable populations, which are constituted generally by random mating of 6–10 inbred lines or progenies of suitable populations. Hybrids are highly heterozygous but genetically homogeneous and phenotypically uniform cultivars which are constituted by crossing two distantly related lines. Improved OPVs of pearl millet are cultivated in about 800,000 ha, mostly in the marginal areas of India (3). OPVs have broad genetic base and are thus, less vulnerable to major diseases by horizontal resistance. The development of OPVs is also very important for deriving inbreds and breeding lines through series of selfing and selection that can be used for hybrid development.

Dietary deficiency of iron (Fe) and zinc (Zn) has been reported to be a grave public health problem, affecting more than two billion people worldwide, especially those in the developing countries (4, 5). Therefore, ending all forms of hunger (including hidden hunger), is one of the agenda of the United Nation's Sustainable Development Goals by 2030 (6). Pearl millet is a nutritious cereal with higher contents of protein, more balanced amino acid profile, and higher levels of micronutrients than those found in other cereals (7–9). Pearl millet is found to be more nutritious than other cereals, however, most of the cultivars grown by the farmers contain only very low levels of micronutrients. The lack of Fe/Zn-rich cultivars/hybrids for cultivation is a major lacuna among all pearl millet growers. Minnis-Ndimba et al. (10) studied the spatial distribution of micronutrients in the grains of pearl millet and showed that both Fe and Zn are predominantly concentrated in the germ, consisting of scutellum, embryo and in the seed coat, which also includes the pericarp and aleurone. Recent studies in pearl millet indicated that there is no significant difference in Fe bioavailability (7.0–7.5%) between the biofortified and the non-biofortified cultivars. However, biofortified varieties contain greater amounts of total iron and zinc densities than the non-biofortified varieties resulting in higher intake of these micronutrients in humans (11, 12). This marks the significant contribution of biofortification to tackle these micronutrient deficiencies in the population dependent on this nutritious cereal. However, the per capita consumption of pearl millet has declined significantly from 11.5 kg (during 1972) to 3.06 kg (by 73.4% in 2010) in rural areas and from 4 kg (during 1972) to 1.13 kg (by 71.8% in 2010) in urban India (13). Considering the fact that pearl millet continues to be an important food crop for India and Africa, creating awareness among the public about the nutritional values of pearl millet is necessary, and biofortification adds the value for its endorsement. The contribution of pearl millet to the total nutrient intake (especially Fe and Zn) from all foods/recipies varies widely across rural India. However, in some parts of rural India (Rajasthan, Maharashtra and Gujarat), the contribution of pearl millet in the intake of micronutrients (Fe and Zn) is very high by 30–50% (14). In a recent initiative by the HarvestPlus Challenge Program, the development of crop cultivars, including pearl millet, with high levels of iron and zinc holds much importance in addressing this issue. Results of a study under this project showed larger variability for Fe and Zn density in pearl millet populations, including some of the officially released OPVs (15, 16). ICTP 8203, an OPV released in India in 1988 (17) and under cultivation since then had been found to have the highest level of Fe and Zn density (18). During the course of inbreeding and selection to develop high-iron potential restorer lines from this OPV, 11 S_3_ progenies were recombined to develop its better version with higher-Fe version. This improved version, designated as ICTP 8203 Fe 10-2, had 9% higher Fe and 11% more grain yield than ICTP 8203 in multi-location trials. It was officially released and adopted as *Dhanashakti* for cultivation in the country (19). Thus, as a fast-track approach, three of high-Fe OPVs were selected to test the effectiveness of intra-population selection for further increasing the Fe and Zn densities. Recurrent selection programs generally follow S_1_ and S_2_ progeny testing and very rarely, if ever, go for S_3_ progeny testing for yield traits. The S_1_ progeny selection has been more frequently used than S_2_ progeny selection because of the necessity of one additional generation per cycle in the latter approach. Earlier studies compared S_1_ and S_2_ selection methods with full-sib (FS) and half-sib (HS) selection methods for grain yield in pearl millet, based on only one cycle of selection. Results showed that S_1_ method is more effective than FS or HS methods (20). In the present study, primary emphasis in S_1_ progeny testing for improved version of the OPV was given to Fe density *per se*. Further improved versions were developed by primarily considering the agronomic scores and also considering their levels of iron. Thus, the objective of this research was to assess the magnitude of variability and test the effectiveness of S_1_ progeny-based genetic improvement in three OPVs of pearl millet for Fe/Zn density.

## Materials and Methods

### Experimental Materials

The basic material for this study consisted of three populations, namely, ICMV 221, AIMP 92901 and ICMR 312. ICMV 221 is an open-pollinated variety (OPV) that was developed at ICRISAT by recombining 124 S_1_ progenies derived from a Bold-seeded Early Composite (BSEC). It was released and notified in 1993 for cultivation at national level in India (21). AIMP 92901 is an OPV that was constituted by recombining 272 S_1_ progenies derived from BSEC and was jointly released by Marathwada Agricultural University and ICRISAT (22). ICMR 312 is an OPV that was developed by recombining 200 S_1_ progenies derived from (BSEC TCP2 C3) and used as a pollinator of a top cross hybrid ICMH 312 (23). All these three OPVs are based on *iniadi* germplasm, which is typically characterized by mostly globular large grains of gray to deep gray color (24). A preliminary trial conducted at ICRISAT showed a few OPVs and breeding lines with higher iron were either based on iniadi germplasm or had a greater percentage of their genes derived from this germplasm (15, 16). Since, AIMP 92901 and ICMR 312 are also based entirely on *iniadi* germplasm; these were included in present study. ICMV 221 was planted in the rainy season of 2008, while AIMP 92901 and ICMR 312 were planted in the summer season of 2009. Each population was planted in 12 rows of 4 m length. The main panicles of about 120 plants in each population were selfed at the boot leaf stage to produce S_1_ progenies for field trials.

### S_1_ Progeny Evaluation and Grain Micronutrient Analysis

The S_1_ progenies of ICMV 221 (97), AIMP 92901 (96), and ICMR 312 (106) where seed was adequate to conduct replicated progeny trial and also for subsequent use in random matting to produce C_1_ bulks, were planted as three separate experiments in adjacent strips in single row plots of 2-m length and replicated twice in randomized complete block design during the rainy season of 2010 in Alfisols at ICRISAT, Patancheru. The planting was done on ridges 75 cm apart. The over-planted rows were weeded and thinned on the 15th day from planting, leaving single plants that are spaced 10 cm apart. Pre-planting application of DAP (Diammonium phosphate, contains 18%N: 46%P) @ 100 kg ha^−1^ was followed with side dressing of urea (46%N) @ 100 kg ha^−1^after thinning.

The progenies were visually assessed for agronomic performance on 1–5 scale (1 poor and 5 best) at or after physiological maturity. Seed set is known to affect Fe and Zn density (25) and inbreeding leads, depending on genotypes and environments, to variable seed set in selfing-derived progenies in pearl millet. Therefore, the progenies were also visually assessed for seed set percentage. As none of the progenies had < 50% seed set, these were scored based on open-pollinated panicles for four broad categories of seed set (50–60% average, 60–75% above average, 75–90% good, and >90% excellent). No xenia and significant dust contamination effect on open-pollinated grains have been reported in pearl millet, and such grains have been found to provide reliable samples for the analysis of Fe and Zn density (25). Therefore, open-pollinated panicles from 6 to 8 random plants were harvested at or after physiological maturity, sundried for 15–20 days, and hand threshed to produce 30–40 g grain samples for Fe and Zn analysis.

**Figure 1:**
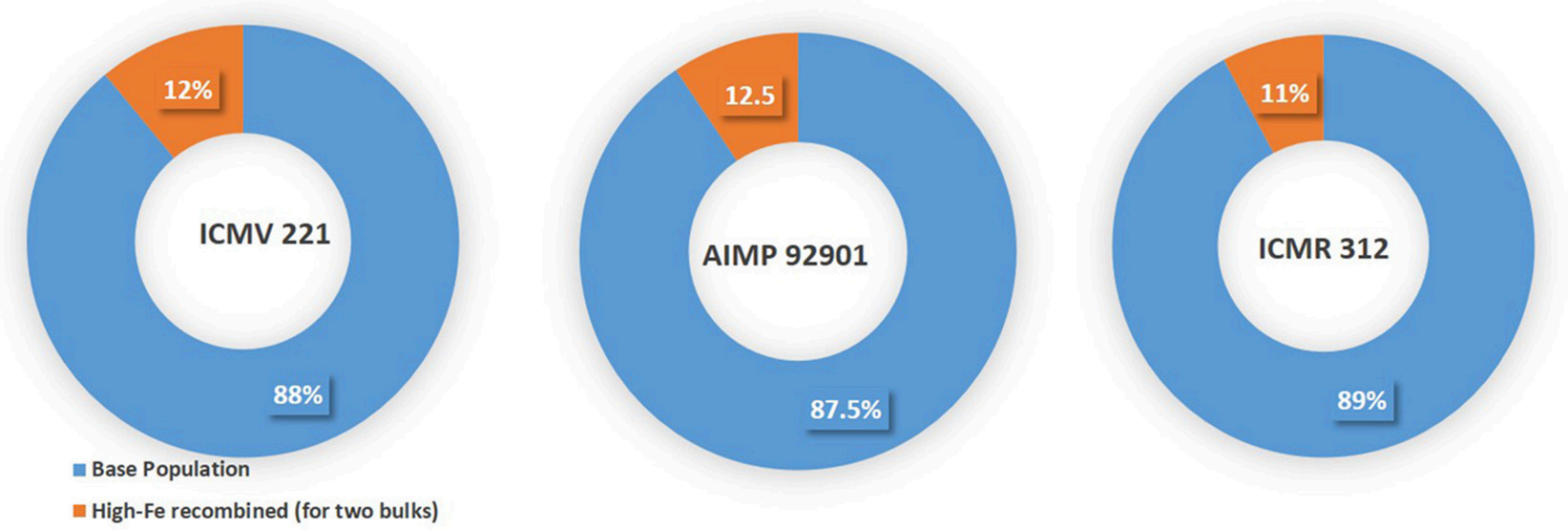
Proportion of S_1_ progenies used in each OPV to derive improved version for Fe and Zn density as biofortified varieties.

The whole grain samples were analyzed using Energy-Dispersive X-ray Fluorescence (XRF) Spectrometer, calibrated and validated for pearl millet at Flinders University, Adelaide, Australia (26). In the above non-destructive XRF analysis, the calibration of Oxford Instruments X-Supreme 8000 fitted with a 10 place auto-sampler was done. About twenty reference whole grain samples of pearl millet that had wide range of ICP-determined Fe (29–163 mg kg^−1^) and Zn (35–100 mg kg^−1^) density were used to calibrate XRF method. Thus, ICP concentrations which were used as reference values were entered into the machine before each sample was scanned. Clean Poly-4 imported film was used for each sample. According to the manufacturer, the X-Supreme 8000 scans a circle of 21 mm diameter with the sample spinner on. All X-Ray scans in this study were performed in this mode, so the scanned area was 346 mm^2^ (26). So, background scans fixed uniform emission toward sampling compartment with 60 s acquisition times for each sample cup to determine micronutrients and expressed in mg kg^−1^. The correlation between XRF and Inductively Coupled Plasma Optical Emission Spectroscopy (ICP) values for Fe and Zn density was highly positive and highly significant (mostly *r* = 0.90; *P* < 0.001). The differences between these two methods were 4–6 mg kg^−1^ for both micronutrients. Based primarily on high Fe density, but also taking seed set and agronomic score into account, 11 progenies in ICMV 221, 10 in AIMP 92901 and 9 in ICMR 312 were selected for recombination to constitute the first improved version of C_1_ bulks (Figure 1; Tables S1, S3, S5). Further, a set of 7 progenies from each OPV, selected principally on the basis of high agronomic score. By also considering high-Fe density, these progenies were used for recombination and the development of the second version of C_1_ bulks (Tables S2, S4, S6). Six progenies in ICMV 221, 5 in AIMP 92901 and 4 in ICMR 312 were common in both the sets.

### Population Bulk Trial and Grain Micronutrient Analysis

The selected progenies for constituting each version of C_1_ bulk populations were planted during the summer season of 2011 in 2 row plots of 4 m length on ridges 60 cm apart, maintaining 10 cm plant-to-plant spacing within the rows. In each plot about 45–50 bagged plants were bagged at the boot leaf stage to avoid contamination from foreign pollen. Pollen was collected from bagged plants in each plot, and the composite pollen bulks were prepared from progenies selected for each version of C_1_ bulk. These were crossed onto 8–10 plants in each plot corresponding to the appropriate version. This process continued for 3–4 days, and about 25–30 plants were crossed in each plot. At or after physiological maturity, all crossed panicles were harvested in each plot, sundried for 15–20 days, and bulk threshed. Equal amounts of seeds form each plot was pooled to produce the improved version of C_1_ bulks of each OPV. The C_0_ bulk (original) and improved versions of C_1_ bulks were planted in a three-replicate trial in randomized complete block design during the summer and rainy seasons of 2012, and summer season of 2013 in Alfisols at Patancheru.

All these plots were planted in 4 rows of 4 m length, with a spacing of 75 cm between rows in rainy season and 60 cm between the rows in the summer. Days to 50% flower was recorded when the main panicles of 50% plants in a plot had fully emerged stigmas. Plant height and panicle length was recorded on 10 random plants in each plot in each replication. About 15 open-pollinated main panicles from each plot were harvested at or after maturity, sun dried for 15–20 days and bulk threshed to produce grain samples. These grain samples were analyzed for Fe and Zn density at the Waite Analytical Services Laboratory, University of Adelaide, Australia, using Inductively Coupled Plasma Optical Emission Spectroscopy (Spectro Analytical Instruments, Kleve, Germany) as described by Wheal et al. (27). In this destructive method, grain samples were oven-dried overnight at 85°C prior to digestion, grounded enough to pass through 1 mm stainless steel sieve using Christie and Norris hammer mill and stored in screw-top polycarbonate vials. The samples were digested with di-acid (Nitric/Perchloric acid) mixture. After digestion, the volume of the digest was made to 25 mL using distilled water; and the content was agitated for 1 min by vortex mixer. The digests were filtered and the Fe concentration was read at 259.94 nm and Zn concentration at 213.86 nm using ICP-OES and these micronutrients were expressed as mg kg^−1^. Care was taken at each step to avoid any contamination of the grains with dust particles and any other extraneous matter (28). A random sample of 200 grains from each plot was weighed and multiplied by factor 5 to determine the thousand grain weight. The weight of grains from these 15 panicles were added to the grain weight of remaining panicles of each plot to calculate the grain yield per ha.

### Statistical Analysis

The S_1_ as well as the population bulk trials were analyzed assuming fixed model following Gomez and Gomez (29) and using Generalized Linear Model procedures (GLM) in Statistical Analysis Systems (SAS) version 9.3 (30). The selection differential (S) was estimated as a deviation of the mean value of the progenies selected as parents for recombination from the mean value of all the progenies included in the S_1_ progeny trials (31). Analysis of variance of the population bulk trial was done with population bulks nested within the OPVs following Gomez and Gomez (29) and further partitioned for each OPV.

## Results and Discussion

Pearl millet is a highly out-pollinated crop. Such a reproductive biology facilitate the production of two distinct cultivars, namely, open-pollinated varieties (OPVs) and hybrids. Although hybrids occupy 60% of cultivated area in India, they have not still reached the semi-arid zones of India and the entire Sub-Saharan Africa. Therefore, the development of OPVs as well as hybrids is equally important for the Global Pearl Millet Improvement Program in ICRISAT. Biofortification is one of the new approaches in plant breeding which symbolizes a marriage of profitable cultivation and improved nutrition in primary cereal crops. Pearl millet is a nutritious crop compared to other major cereals but not all the farmers are able to grow varieties or hybrids with improved nutritional or protein profiles. The number of popular cultivars which are rich in Fe/Zn is almost negligible. Unlike the breeding methods for improving yield traits, the methods for utilizing the variability for micronutrients present in the germplasm has not yet been exploited thoroughly. For instance, the average densities of Fe (42 mg kg^−1^) and Zn (32 mg kg^−1^) in commercially grown hybrids in India are very low. This is lower than the baseline for Fe density (47 mg kg^−1^) set by the HarvestPlus Program (16). The total genetic variability for the targeted essential micronutrients (Fe and Zn) in the germplasm pool is revealed by means of nutritional breeding. The fast-track breeding procedure involves the existing commercial OPVs. OPVs have large amounts of trait diversity within acceptable maturity range. This opens up opportunity to exercise progeny selection (for any traits) and to exploit the available variability whereas hybrids, bred for uniformity will not have variability for micronutrients, as these traits are not even monitored in due course of breeding their parental lines. Considering the above facts, the present study aimed to demonstrate the effectiveness of a fast-track breeding approach that uses intra-population variability, in commercial OPVs to enhance the Fe and Zn densities and develop their essentially-derived versions. For the interest of research community and relevance of this study, the results and discussion are combined hereunder.

The OPV ICMV 221 showed significant genetic variability with four-fold difference in Fe density among the S_1_ progenies, which range from 31 to 117 mg kg^−1^ (Table 1). One progeny had exceptionally high Fe density of 143 mg kg^−1^, which could have resulted from the seed sample and/or the analytical tool used for the analysis. This specific progeny with this high Fe density had only above-average seed set, and reduction in seed set has been shown to be associated with the overestimation of Fe density in pearl millet (25). Based on the results of several trials, it has also been observed that XRF may occasionally overestimate Fe density (32). Amongst the three OPVs, the progenies of ICMV 221 C_0_ bulk had the highest mean Fe density of 75 mg kg^−1^, and the progenies selected for constituting the two C_1_ bulks had 32–33 mg kg^−1^ of Fe, as selection differentials (SD). The Fe density in the progenies of AIMP 92901 ranged from 40 to 104 mg kg^−1^, with a mean of 68 mg kg^−1^. Whereas, the selection differential was 23 mg kg^−1^ in the full set of progenies (11) to constitute its first C_1_ bulk and 26 mg kg^−1^in the second set (7 progenies) selected to constitute the second C_1_ bulk. The S_1_ progenies of ICMR 312 had about three-fold difference for Fe density, varying from 34 to 96 mg kg^−1^with a mean value of 65 mg kg^−1^ and selection differentials of 22–23 for the progenies selected to constitute the two C_1_ bulks. About two-fold differences were observed among the S_1_ progenies for Zn density in all three populations. The selection differentials of the progenies selected to constitute the two versions of C_1_ bulks were 12–13 mg kg^−1^ for ICMV 221, 8–10 mg kg^−1^for AIMP 92901, and 7 mg kg^−1^ for ICMR 312. Clearly, the selection differentials of the progenies selected to constitute the two versions of C_1_ bulks were not different either for Fe or Zn density in all three OPVs because the second set of progenies (in each OPV) included about 50% of those included in the first set.

**Table 1:** Mean and range among S_1_ progenies tested, and selection differential (SD) for Fe and Zn densities in selected progenies of pearl millet, rainy season (2010), Patancheru.

**Population bulk**	**No. of progenies**	**Fe density (mg kg**^****−1****^**)**			**Zn density (mg kg**^****−1****^**)**		
		**Range**	**Mean**	***S*D**	**Range**	**Mean**	***SD***
ICMV 221-C0	97	31–143	75	–	36–82	59	–
ICMV 221-Fe 11-1	11	94–143	108	33	63–80	72	13
ICMV 221-Fe 11-2	7	94–143	107	32	63–78	71	12
AIMP 92901-Fe-C0	96	40–104	68	–	35–79	58	–
AIMP 92901-Fe 11-1	10	84–102	91	23	57–79	68	10
AIMP 92901-Fe 11-2	7	84–102	94	26	58–79	66	8
ICMR 312-C0	106	34–96	65	–	35–69	52	–
ICMR 312-Fe 11-1	9	81–96	88	23	50–69	59	7
ICMR 312-Fe 11-2	7	81–96	87	22	52–65	59	7
SE±			2.1			1.4	

*SE, Standard error of mean*.

The mean grain yield in population bulk trial varied from 3.3 t ha^−1^ in the summer season of 2013 to 5.1 t ha^−1^ in the summer season of 2012, indicating large productivity level differences in the environments (Table S7). The Fe density across the environments varied from 64 mg kg^−1^ in the summer season of 2012 to 80 mg kg^−1^ in the rainy season of 2012; and the Zn density varied from 53 to 61 mg kg^−1^ under the same seasonal conditions. The difference among the population bulks of ICMV 221 was highly significant (*P* < 0.01), both for Fe and Zn density (Table 2). The differences were also highly significant (*P* < 0.01) for Fe density in ICMR 312, and significant for Zn density (*P* < 0.05) in AIMP 92901. Population × environment interaction was not significant for micronutrients and other traits except days to flowering in each individual and across OPVs. Thus, genotype × environment interaction for these micronutrients did not result in any change in the relative ranking of different population across the environments. Similar results have been reported in pearl millet studies for Fe and Zn densities (33, 34) and Cassava for carotenoid contents (35). The first version C_1_ bulk of ICMV 221 (ICMV 221 Fe 11-1) had the mean Fe density of 76 mg kg^−1^ (21% higher than the C_0_ bulk), and the second version C_1_ bulk had 70 mg kg^−1^ Fe density (11% higher than the C_0_ bulk) (Table 3). The Zn density in these two C_1_ version bulks, respectively, was 61 mg kg^−1^ (15% higher than the C_0_ bulk), and 58 mg kg^−1^ (9% higher than the C_0_ bulk). There were highly significant differences among the population bulks of ICMV 221 for days to 50% flowering and plant height, with the C_1_ bulks being 3–4 days later to flowering and 8–13 cm taller in height.

**Table 2:** Mean square for grain iron (Fe) and zinc (Zn) densities, grain yield ,and agronomic traits across three seasons in population bulks of three OPVs in pearl millet, Patancheru.

**Source**	**DF**	**Mean square**						
		**Fe (mg kg^**−1**^)**	**Zn (mg kg^**−1**^)**	**Grain yield (kg ha^**−1**^)**	**Days to 50% flowering (d)**	**Plant height (cm)**	**Panicle length (cm)**	**1000-grain weight(g 1000^**−1**^)**
Environment (E)	2	1745.0^**^	255.7^**^	23,857,604^**^	413.9^**^	16,032^**^	31.0^**^	17.1^**^
Replication/E	6	72.1	25.4	2,30,195	1.1	63.1	6.1^**^	2.2
Bulk	8	268.3^**^	88.7^**^	1,64,068	41.8^**^	171.2^**^	5.3^**^	4.1^**^
Population	2	336.7^**^	97.7^**^	2,14,620	139.3^**^	188.5^*^	8.8^**^	9.8^**^
Bulk/population	6	245.4^**^	85.7^**^	1,47,217	9.2^**^	165.5^**^	4.1^*^	2.2
ICMV 221 bulk	2	406.2^**^	151.5^**^	2,68,140	25.4^**^	452.5^**^	1.3	2.8
AIMP 92901 bulk	2	125.5	73.2^*^	1,61,548	2.1	4.9	9.1^**^	2.2
ICMR 312 bulk	2	204.6^*^	32.4	11,965	0.1	39.0	2.0	1.7
Bulk × E	16	65.6	22.2	96,542	7.1^**^	59.0	1.5	0.9
Population × E	4	73.6	34.7	2,23,513	17.6^**^	110.1^*^	2.0	0.9
Bulk/population × E	12	62.9	18.1	54,218	3.6^**^	42.0	1.4	0.9
ICMV 221 bulk × E	4	45.3	6.8	1,01,239	5.9^**^	81.8	0.3	1.6
AIMP 92901 bulk × E	4	30.7	16.7	31,598	4.7^**^	7.4	2.6	0.4
ICMR 312 bulk × E	4	112.8	30.8	29,818	0.3	36.8	1.2	0.8
Error	48	39.6	16.6	1,91,134	0.9	38.8	1.4	1.3

* *Significant at the 0.05 probability level*.

** *Significant at the 0.01 probability level*.

**Table 3:** Grain iron (Fe) and zinc (Zn) densities, grain yield and agronomic traits in population bulks of three OPVs in pearl millet, mean of three seasons at Patancheru.

**Population bulk**	**Fe (mg kg^**−1**^)**	**Zn (mg kg^**−1**^)**	**Grain yield (kg ha^**−1**^)**	**Days to 50% flowering (d)**	**Plant height (cm)**	**Panicle length (cm)**	**Grain weight (g 1000^**−1**^)**
ICMV 221-C0	63	53	3,978	39	165	22	14.2
ICMV 221-Fe 11-1	76	61	3,914	42	173	23	15.3
ICMV 221-Fe 11-2	70	58	4,240	43	178	22	14.7
ICMR 312-C0	63	52	4,161	46	176	24	13.1
ICMR 312-Fe 11-1	70	53	4,200	45	178	24	13.5
ICMR 312-Fe 11-2	72	56	4,234	46	177	23	14
AIMP 92901-Fe-C0	59	52	4,335	43	176	25	14.3
AIMP 92901-Fe 11-1	66	57	4,067	42	172	22	13.7
AIMP 92901-Fe 11-2	64	56	4,190	43	174	22	14.6
LSD (5%)^€^	6	4	414	1	6	1	1.1

€ *Least significant difference*.

The Fe density of the first C_1_ bulk of ICMR 312 was 70 mg kg^−1^ (11% higher than the Co bulk) and that of the second C_1_ bulk was 72 mg kg^−1^mg kg^−1^ (14% higher than the C_0_ bulk). Although the difference among the population bulks of AIMP 92901 for Fe density was not significant, the first C_1_ version had 66 mg kg^−1^ Fe density (12% higher than the C_0_ bulk). It also had 57 mg kg^−1^ Zn density (10% higher than the C_0_ bulk). There were no significant differences among the population bulks either in ICMR 312 or in AIMP 92901 for grain yield, time of flowering, plant height, panicle length and seed weight except that the panicles of the C_1_ bulks of AIMP 92901 were significantly shorter by 3 cm. It was also found in ICMV 221, that the population bulks did not differ significantly for grain yield, panicle length and 1,000-seed weight.

The above results indicate that the selection for Fe density was effective in all three OPVs, and the magnitude of selection response largely depended on the magnitude of genetic variability and selection differentials. Thus, the largest selection response was observed in ICMV 221, which, among the three OPVs, had the largest variability and the highest selection differential for Fe density. This population also registered significant and the largest selection response for Zn density, which is not unexpected, considering the highly significant and high positive correlation (*r* = 0.70, *P* < 0.01) between Fe and Zn densities observed in the S_1_ progeny trial of this OPV. Earlier studies in pearl millet have all shown highly significant and positive correlation between Fe and Zn densities, with the magnitude of correlation coefficient being dependent on the genetic materials used and the environment (15, 18, 25, 36–40). As compared to ICMV 221, the correlation coefficients between Fe and Zn density, although highly significant, were relatively low, both in ICMR 312 (*r* = 0.52; *p* < 0.01) and AIMP 92901 (*r* = 0.47; *p* < 0.01). The selection differentials for Fe and Zn densities in these two OPVs were also less than those observed in ICMV 221. Thus, selection response for Fe density was lower in these two populations, as compared to that observed in ICMV 221, the selection response for Zn density was also lower.

In the present study, a second version of C_1_ bulks was constituted primarily based on the visual assessment of agronomic score (that included yielding ability) but also taking the Fe density into account. Nonetheless, its yield did not differ significantly from the yield of C_0_ bulk in all three OPVs. This ineffectiveness of selection for grain yield could have resulted from the smaller size of the trial plots (of 2 m length) and the minimal number of replications (only twice) used in this study. Another reason for this result could predominantly be the non-additive mode of genetic variability for grain yield as reported in most of the pearl millet studies (39, 41). In contrast, Fe and Zn densities are the measurable traits and have been reported to be predominantly under additive genetic control (33, 38, 39), which could enhance the effectiveness of selection during early generations even in smaller plots. Significant selection response for Fe density and lack of significant difference among the population bulks for grain yield in all three populations indicated that effective selection in populations for Fe density can be made, at least, without compromising on grain yield. For the simultaneous selection for Fe density and grain yield to be effective, one needs to use a trial plot of size of at least 4 m length (normal practice in pearl millet improvement), the genotypes being replicated three times, and exercise moderate selection intensity, of say 30–40%, at the S_1_ stage and delay high intensity selection to the later selfing generations. Following a sequential selection procedure from S_1_ though S_3_ generation for estimation of Fe density and visual assessment of agronomic score on yielding ability, a C_1_ version of a commercial variety ICTP 8203 selected for high Fe density was developed. This high-Fe version had 9% higher Fe density and 11% higher grain yield than the C_0_ version, based on 42 location × year trials in India (19). This result showed that effective simultaneous selection for Fe density and grain yield is possible in pearl millet. Studies in other crops like cassava (42) and maize (43) for carotenoid contents, and strontium (Sr) content in wheat and barley (44) have demonstrated that the mineral contents can be improved considerably between and within the populations.

The selection for Fe density had no significant effects on any of the agronomic traits studied except for maturity as 3–4 days of delayed flowering and 8–13 cm increase in plant height of the C_1_ bulks of ICMV 221, and 3 cm shorter panicles of the C_1_ bulks of AIMP 92901. Inconsistency of these changes across the three populations suggests that these changes occurred either as random events, or were characteristics of specific genetic constitutions of the populations, and may not be a general feature reflecting on the association of Fe and Zn densities with the time of flowering and plant height. For instance, the selected high-Fe version of ICTP 8203 mentioned above had similar flowering time but had plants that were 7 cm taller than the original (C_0_) bulk (19). While one pearl millet study observed significant positive association between Fe density and time to 50% flowering (36), another study showed that both Fe and Zn densities were negatively correlated with plant height, days to flowering and panicle length, although not significant in all the environments (40). These results would indicate that association of Fe and Zn densities with grain yield and other agronomic traits remains a subject meriting further investigations.

## Conclusion

The biofortification of pearl millet is focused on improving the levels of both iron and zinc which are associated to one another. Huge genetic variability for Fe and Zn densities was observed among the S_1_ progenies in all the three OPVs used in this study. Progeny selection within population was effectively demonstrated with high selection response for both the micronutrients (10–21%). Results are in conformity with the previous reports indicating additive genetic control of both micronutrients which is reflected in the effectiveness of progeny test-based population improvement. Increased cycle of re-selection and subsequent recombination to assemble more favorable alleles controlling Fe/Zn may yield greater difference from the original bulks. This study necessitates the worthiness of genomic tools and analyses that can play major role in discovering such alleles and their loci in the original and improved versions. There were very high correlations between Fe and Zn densities and no significant change in the grain yield as compared to the original population, indicating good prospects of simultaneous genetic improvement for both micronutrients without compromising on grain yield. Nevertheless, conscious selection may be equally important for Zn density along with Fe density for greater genetic gain than that observed in this study. These improved populations can be evaluated in multi-location trials to confirm their stability and superior performances. They can be identified as essentially-derived varieties for higher micronutrients levels with no changes in grain yield and agronomic traits.

## Author Contributions

MG and KR designed the study and prepared the final manuscript. AK and AR analyzed the data and prepared initial draft. HS managed the field trials and data collection. All of the authors have read and approved this manuscript and was involved in the field experiments.

### Conflict of Interest Statement

The authors declare that the research was conducted in the absence of any commercial or financial relationships that could be construed as a potential conflict of interest.

## Abbreviations

AIMP: Aurangabad/ICRISAT millet population
ICMR: ICRISAT millet restorer
ICMV: ICRISAT millet variety
OPVs: Open-pollinated varieties
XRF: X-ray fluorescence spectrometer.
