# Supplementary material for: Nutritional Security in Drylands: Fast-Track Intra-Population Genetic Improvement for Grain Iron and Zinc Densities in Pearl Millet
Source: Front Nutr. 2019 May 24;6:74. doi: 10.3389/fnut.2019.00074 (PMC6543707; doi:10.3389/fnut.2019.00074)
Supplement: Supplementary file 1 [file Data_Sheet_1.PDF]

Supplementary table

Table S1. Selected ICMV 221 progenies (S1s) based on grain Fe and seed set data for producing ICMV 221 Fe-11-1

Entries	OP- Seed Set class	Days to 50% flower	XRF Fe density	XRF Zn density
ICMV 221 S1 - 146	A	45	143	63
ICMV 221 S1 - 175	G	44	117	75
ICMV 221 S1 - 107	G	46	114	78
ICMV 221 S1 - 117	G	44	108	73
ICMV 221 S1 - 120	>A	43	108	76
ICMV 221 S1 - 138	G	44	107	63
ICMV 221 S1 - 84	G	44	104	80
ICMV 221 S1 - 269	G	43	98	77
ICMV 221 S1 - 174	G	42	95	66
ICMV 221 S1 - 296	>A	44	95	67
ICMV 221 S1 - 360	E	45	94	73

Seed set class: 50-60% Average (A), 60-75% Above Average (>A), 75-90% Good (G), >90% Excellent (E)

Table S2. Selected ICMV 221 progenies (S1s) based on grain Fe and seed set data for producing ICMV 221 Fe-11-2

Entries	OP- Seed Set class	Days to 50% flower	XRF Fe density	XRF Zn density
ICMV 221 S1 - 146	A	45	143	63
ICMV 221 S1 - 107	G	46	114	78
ICMV 221 S1 - 117	G	44	108	73
ICMV 221 S1 - 269	G	43	98	77
ICMV 221 S1 - 296	>A	44	95	67
ICMV 221 S1 - 373	A	44	95	67
ICMV 221 S1 - 360	E	45	94	73

Seed set class: 50-60% Average (A), 60-75% Above Average (>A), 75-90% Good (G), >90% Excellent (E)

Table S3. Selected AIMP 92901 progenies (S1s) based on grain Fe and seed set data for producing AIMP 92901 Fe-11-1

Entries	OP- Seed Set class	Days to 50% flower	XRF Fe density	XRF Zn density
AIMP 92901-S1-122	A	44	102	58
AIMP 92901-S1-278-1	G	50	98	66
AIMP 92901-S1-143	A	42	94	63
AIMP 92901-S1-87	G	46	93	79
AIMP 92901-S1-248	G	52	92	57
AIMP 92901-S1-162	G	45	90	65
AIMP 92901-S1-68	>A	43	87	70
AIMP 92901-S1-120	G	47	86	79
AIMP 92901-S1-291-2	A	58	86	65
AIMP 92901-S1-5-2	G	50	85	60

Seed set class: 50-60% Average (A), 60-75% Above Average (>A), 75-90% Good (G), >90% Excellent (E)

Table S4. Selected AIMP 92901 progenies (S1s) based on grain Fe and seed set data for producing AIMP 92901 Fe-11-2

Entries	OP- Seed Set class	Days to 50% flower	XRF Fe density	XRF Zn density
AIMP 92901-S1-122	A	44	102	58
AIMP 92901-S1-278-1	G	50	98	66
AIMP 92901-S1-160	G	47	97	72
AIMP 92901-S1-143	A	42	94	63
AIMP 92901-S1-87	G	46	93	79
AIMP 92901-S1-162	G	45	90	65
AIMP 92901-S1-6	G	50	84	62

Seed set class: 50-60% Average (A), 60-75% Above Average (>A), 75-90% Good (G), >90% Excellent (E)

Table S5. Selected ICMR3 12 progenies (S1s) based on grain Fe and seed set data for producing ICMR 312 Fe-11-1

Entries	OP- Seed Set class	Days to 50% flower	XRF Fe density	XRF Zn density
ICMR 312 S1-126	E	50	96	61
ICMR 312 S1-131	A	48	90	69
ICMR 312 S1-184-2	G	55	90	57
ICMR 312 S1-113	>A	47	89	61
ICMR 312 S1-17-1	G	52	89	52
ICMR 312 S1-209	G	49	87	50
ICMR 312 S1-296	A	44	86	64
ICMR 312 S1-106	G	47	82	60
ICMR 312 S1-250	G	43	81	55

Seed set class: 50-60% Average (A), 60-75% Above Average (>A), 75-90% Good (G), >90% Excellent (E)

Table S6. Selected ICMR3 12 progenies (S1s) based on grain Fe and seed set data for producing ICMR 312 Fe-11-2

Entries	OP- Seed Set class	Days to 50% flower	XRF Fe density	XRF Zn density
ICMR 312 S1-126	E	50	96	61
ICMR 312 S1-184-2	G	55	90	57
ICMR 312 S1-17-1	G	52	89	52
ICMR 312 S1-85	>A	45	88	65
ICMR 312 S1-112	G	44	85	58
ICMR 312 S1-136	G	50	83	65
ICMR 312 S1-250	G	43	81	55

Seed set class: 50-60% Average (A), 60-75% Above Average (>A), 75-90% Good (G), >90% Excellent (E)

Table S7: Field performance for gain iron (Fe) and zinc (Zn) densities, grain yield and agronomic traits of population bulks of three OPVs in pearl millet, in three crop seasons at Patancheru

Population Bulk	Fe (mg kg^{-1})			Zn (mg kg^{-1})			Grain yield (kg ha^{-1})			Days to 50% flower (d)			Plant height (cm)			Panicle length (cm)			Grain weight (g 1000^{-1})		
	E1	E2	E3	E1	E2	E3	E1	E2	E3	E1	E2	E3	E1	E2	E3	E1	E2	E3	E1	E2	E3
ICMV 221 C0	61	69	59	51	58	51	4808	4009	3115	41	39	38	166	189	134	23.5	21.3	20.9	13.5	14.8	14.3
ICMV 221 Fe-11-1	70	88	72	58	64	62	4758	3868	3115	46	41	39	185	188	146	24.2	21.3	21.8	15.5	16.3	14.0
ICMV 221 Fe-11-2	64	85	62	53	63	57	5401	3934	3384	47	41	40	182	199	149	23.8	21.9	22.0	15.3	15.3	13.6
ICMR 312 Co	63	64	62	52	50	55	5080	3951	3453	52	43	42	190	186	149	25.4	22.8	22.7	12.8	13.5	13.1
ICMR 312 Fe-11-1	73	76	60	52	56	51	5101	3963	3535	51	42	43	193	193	148	24.8	23.9	22.6	13.3	14.5	12.7
ICMR 312 Fe-11-2	64	82	71	52	58	58	5283	3822	3597	52	43	42	183	190	149	24.1	23.4	21.2	13.1	15.4	13.4
AIMP 92901 C0	54	68	54	49	57	49	5661	3966	3378	48	42	39	184	192	147	23.7	24.5	23.5	13.3	15.4	14.2
AIMP 92901 Fe-11-1	58	76	64	52	60	59	5235	3787	3181	45	42	39	182	194	146	24.3	22.4	21.6	13.0	14.5	13.5
AIMP 92901 Fe-11-2	58	79	56	53	61	53	5335	3819	3418	46	41	41	185	196	145	22.9	20.9	21.9	14.2	15.9	13.8
LSD (5%)	13	12	5	7	8	6	1107	545	442	2	1	2	13	10	9	1.9	1.7	2.4	2.2	2.0	1.6

LSD-Least Significant difference

E1- 2012 Summer crop season

E2- 2012 Rainy crop season

E3-2013 Summer crop season
